# Hydrogels for Liver Tissue Engineering

**DOI:** 10.3390/bioengineering6030059

**Published:** 2019-07-05

**Authors:** Shicheng Ye, Jochem W.B. Boeter, Louis C. Penning, Bart Spee, Kerstin Schneeberger

**Affiliations:** Department of Clinical Sciences of Companion Animals, Faculty of Veterinary Medicine, Utrecht University, 3584 CT Utrecht, The Netherlands

**Keywords:** hydrogel, tissue engineering, liver, bioengineered organ

## Abstract

Bioengineered livers are promising in vitro models for drug testing, toxicological studies, and as disease models, and might in the future be an alternative for donor organs to treat end-stage liver diseases. Liver tissue engineering (LTE) aims to construct liver models that are physiologically relevant. To make bioengineered livers, the two most important ingredients are hepatic cells and supportive materials such as hydrogels. In the past decades, dozens of hydrogels have been developed to act as supportive materials, and some have been used for in vitro models and formed functional liver constructs. However, currently none of the used hydrogels are suitable for in vivo transplantation. Here, the histology of the human liver and its relationship with LTE is introduced. After that, significant characteristics of hydrogels are described focusing on LTE. Then, both natural and synthetic materials utilized in hydrogels for LTE are reviewed individually. Finally, a conclusion is drawn on a comparison of the different hydrogels and their characteristics and ideal hydrogels are proposed to promote LTE.

## 1. Introduction

Liver tissue engineering (LTE) aims to construct liver models that mimic the functions of an in vivo liver as closely as possible. LTE has two main applications: First, as in vitro models, bioengineered livers can be used for testing of xenobiotics (e.g., drugs and pathogens), toxicological studies and as (patient-specific) disease models [1]. Ethical and practical issues hamper to conduct research on drugs and pathogens with living human beings; on the other hand, in vitro models, either hepatoma cell lines or primary human hepatocytes, cannot represent the true in vivo characteristics, where liver cells are spatially localized and cell polarity provides dynamic cues for cellular activities [2,3]. Thus, LTE could be used for drug development and toxicity testing [4] and as cell models for pathogen testing. Second, although currently far from clinical application, LTE aims to develop alternatives to donor organs for in vivo transplantations. Liver diseases are a major concern as they account for millions of deaths annually and the incidence of liver disease is still increasing worldwide [5]. End-stage liver disease or liver failure is the direct cause of death and the only curative option is orthotopic liver transplantation (OLT) [6,7,8]. However, donor shortage has restricted this treatment severely and many patients die while on the waiting list for applicable donor livers [9,10]. To solve the problem of donor shortage, hopes are that bioengineered livers could be an alternative in the future, and LTE is an essential approach to fabricate bioengineered livers. 

Cell sources and supportive materials are the most fundamental ingredients for LTE. First of all, hepatic cells are indispensable and there are already several possible cell sources [5,7,8]. Primary hepatocytes are typically selected as the cell source [11,12,13,14,15] but are limited by the availability of primary tissue, the difficulty in maintaining the hepatic phenotype, and expanding the cells sufficiently [16,17]. Therefore, stem cells or progenitor cells that differentiate into the hepatic lineage are a viable alternative [18,19,20], and methods to expand induced pluripotent stem cells (iPSCs) or adult stem cell-derived hepatic cells have also been established [21,22,23]. The maturation status and hence function of stem cell derived hepatic cells do not reach primary hepatocyte levels yet, but can presumably be increased in the future by a combination of several maturation approaches [24]. Additionally, several groups have recently developed techniques which now allow for efficient in vitro expansion of primary human hepatocytes [25,26,27]. Now that methods have been developed for long-term culture of cells with hepatocyte function, there is a clear need to optimize biomaterials aiming to assemble various liver cell types properly.

Hydrogels are one of the most promising candidates to serve as supportive biomaterials and have been frequently used in tissue engineering and regenerative medicine (TERM) [28]. There are ample reviews or articles describing a wide variety of hydrogels [29,30,31]. Most of them are only focused on specific biomaterials such as nanocellulose [32], fibrin [33], collagen [34], poly(e-caprolactone) (PCL) [35], and poly(vinyl alcohol) (PVA) [36], and discuss the design methods [37,38] or proposed possible applications in TERM [39,40]. However, there is no clear statement on the different hydrogels used for LTE. Even though great improvements have been achieved, there are still no hydrogels available that mimic liver extracellular matrix (ECM) functionally, restricting LTE for both in vitro models and in vivo transplantation. Here, we compare different hydrogels used in LTE, and suggest possible applications.

## 2. Liver and LTE

### 2.1. Liver Functions and LTE

The main goal of LTE is to recapitulate main liver functions, not necessarily the liver architecture per se. The liver originates from the endoderm in the embryonic foregut [41] and is the largest internal organ in the human body, accounting for 2–5% of the body weight. It performs a complex array of more than 500 functions, including metabolic, synthetic, immunologic, and detoxification processes [8]. The most essential activities of the liver are to maintain an active urea cycle, albumin synthesis and drug metabolism as well as regulating whole-body metabolism and xenobiotic detoxification [42]. The liver has to face challenges daily while performing those vital functions, which may result in diseases caused by toxins, drugs, and viruses [8,9]. In addition, autoimmune diseases and liver cancer occur frequently [8,43]. These diseases can impair liver function and eventually lead to end-stage liver disease. Luckily, the liver has tremendous capacity to regenerate [44]. In the past decades, a comprehensive understanding of the mechanisms of liver regeneration has been established and a dozen reviews [42,44,45,46,47,48,49,50,51,52,53,54] have shown many different aspects of liver regeneration. Nevertheless, in many clinical scenarios liver regeneration is not sufficient to circumvent loss of a large volume of hepatic tissue [55]. LTE can on the one hand provide in vitro models for a better understanding of the pathophysiology of such liver diseases, and thereby contribute to the development of new treatment options. On the other hand, LTE might provide a treatment by itself in the future, and many groups have started to investigate the possibility of LTE for the creation of suitable liver transplants.

### 2.2. Liver Histology and LTE

**Figure 1:**
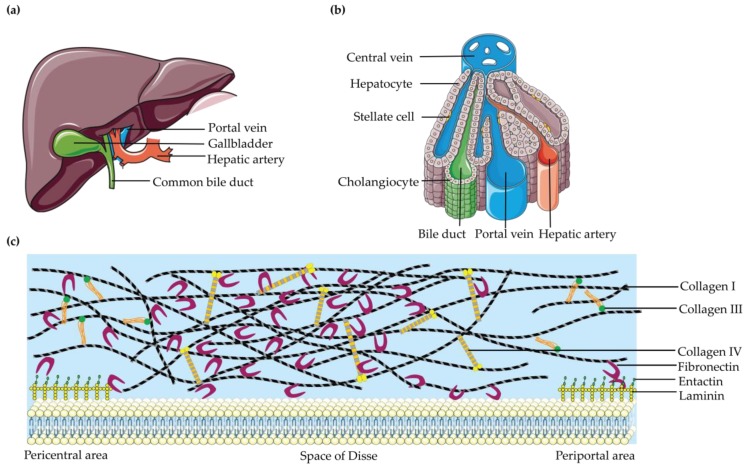
Liver histology and extracellular matrix (ECM). (**a**) A schematic representation of the whole human liver; (**b**) Schematic overview of the liver lobule; (**c**) The connection among major liver ECM components seen within the space of Disse.

The liver is one of the most complex organs in the human body (Figure 1). The mature human liver is composed of four lobes and structurally and histologically, the liver can be divided into four tissue systems [56]: intrahepatic vascular system, stroma, sinusoidal cells, and hepatocytes. Those tissue systems are made from multiple cell types, including the parenchymal cells, hepatocytes, and cholangiocytes, together with various non-parenchymal cells [57,58]. Hepatocytes constitute ~80% of the liver mass. The remaining part is made up by non-parenchymal cells, including liver sinusoidal endothelial cells, Kupffer cells, lymphocytes and stellate cells [44,59]. Although they take up a small portion of the liver volume (6.5%), they constitute 30–40% of the total cell number [6]. Those cell types enable the liver to exhibit a hierarchical structure consisting of repeated functional tissue units, the liver lobules. Within a lobule, a smaller amount of oxygenated blood enters through branches of the hepatic artery and the largest amount of low oxygenated blood enters through the portal vein and flows in specialized sinusoidal vessels toward the central vein. Bile, which is produced and excreted by hepatocytes, flows in the opposite direction towards the intrahepatic bile duct. Hepatocytes are polarized epithelial cells that interact closely with a number of nonparenchymal cell types along the sinusoidal tracts of the liver lobule. Collectively, these cellular components and multiscale tissue structures contribute to the diverse functional roles of the liver [8]. 

Depending on the application of a bioengineered liver, it might not be necessary to recapitulate this entire complexity of the liver in LTE. For example, to study the pathophysiology of alpha1-antitrypsin (A1AT) deficiency, a purely epithelial liver model containing hepatocyte-like cells seems sufficient [23]. In general, however, a close-to-physiological 3D organization, cell composition and ECM has been shown to significantly improve the maturation and function of bioengineered tissues [24]. Most importantly, the cellular interactions [8] of the liver have to be established in order to create a structure that is similar to the native liver in both mechanism and function.

### 2.3. Liver ECM and LTE

Mimicking the liver ECM is another indispensable constitution for LTE. Although the ECM is only a small component of the liver, less than 3% of the relative area on a normal liver section [60], it has a crucial role [61]. The ECM provides cohesiveness within tissue compartments, induces polarization of cells, and acts as a major determinant of gene expression and differentiation [62,63]. As the major component of stroma [6], the liver ECM, mainly located at the interface between the blood flow and the epithelial compartment, plays a vital role in supporting and connecting hepatic cells, and also fulfills a big role in the polarity of parenchymal cells and thus the liver function. There are differences among ECM distributions of different areas in the adult liver. The liver can be divided into four major compartments: capsule, portal spaces, lobular interstitium (subsinusoidal space or space of Disse), and central space. The unique nature of the liver ECM is seen in the special configuration of the space of Disse. The liver lobule has no basement membrane (BM) and only an attenuated ECM consisting mostly of fibronectin, some collagen type I, and minor quantities of collagen types III, IV, V, and VI [61]. The structure and composition varies greatly in diseased livers [64,65,66,67]. Under normal conditions, the liver ECM consists of collagens type I and III (large fibrils), IV (net structure), V and VI (small fibrils), glycoproteins (laminin and fibronectin), elastins, glycosaminoglycans, and proteoglycans [68]. In fibrotic liver the ECM components are similar to those present in normal liver but are quantitatively increased (three- to five-fold increase in ECM) [64]. When liver damage is present, the liver ECM is produced mainly by hepatic stellate cells [6], the major fibrogenic cell type in human liver [69]. Even though fibrous tissue is quantitatively very limited in liver [64], the liver ECM forms the fibrous scaffold, provides a surface for cell adhesion, space for cell growth and migration, interacts with liver progenitor cells [70], and consists mostly of fibronectin, laminins, collagens, and signaling molecules [65,67]. Therefore, any modification in the liver ECM has a direct effect on liver structure and functions [64,71], which underlines the importance to mimic the liver ECM in LTE.

## 3. Hydrogels for LTE

Hydrogels are a promising candidate to mimic the liver ECM functionally in LTE. A hydrogel is a network of natural or synthetic hydrophilic polymer chains possessing a degree of flexibility similar to natural tissues. The term “hydrogel” first appeared in literature in 1894 [72] and the first generation of hydrogels were developed around 1960s, when poly(vinyl alcohol) (PVA) [73] and poly(2-hydroxyethl methacrylate) (pHEMA) [74] were described for the first time in publications. After the development for three generations, hydrogels are progressing to smart materials [75]. In order to find suitable hydrogels for LTE, various materials have been tested, but to date there is no hydrogel that mimics liver ECM adequately. Here, significant properties for hydrogels to mimic the liver ECM are introduced, together with hydrogels frequently utilized for LTE, in order to provide insights into hydrogels for LTE.

### 3.1. Properties Significant for LTE

To make the best use of different hydrogels, comprehensive understanding of their characteristics is necessary to mimic the liver ECM that is responsive for liver cell engraftment, long-term survival and function [76]. Those characteristics determine their various properties and several pivotal properties for LTE have been emphasized in the hope of optimizing the most suitable hydrogels. Properties of the ideal scaffold for LTE have been listed by Vasanthan et al. [77]. Here, those properties are integrated into two basic groups: biological properties [78] and physicochemical properties.

#### 3.1.1. Biological Properties

The most fundamental characteristics of hydrogels for LTE are appropriate biological properties. Biological properties, such as biocompatibility [79,80], biodegradability, and bioactivity, have always received great attention when a hydrogel is used for TERM [81]. For example, cellular biocompatibility makes the nanofibrillar cellulose hydrogel suitable for the proliferation and differentiation of human hepatic cell lines [82]. Biodegradability makes hydrogels promising in applications on transplantation purposes [83]. Biodegradable hydrogels can not only act as the supportive scaffold for cells to perform many kinds of activities and form desirable tissue, they also provide the possibility to be cleared locally by enzymes that are specific to degrade those biomaterials [81]. The degradation speed can be regulated by the polymerization of the hydrogel.

Nevertheless, biocompatibility and biodegradability is not enough for hydrogels to support liver functions for LTE. They should also be bioactive, which means that the hydrogels are capable of transmitting dynamic signals instantly and are able to perform a variety of stimuli responses properly. To obtain these characteristics, spatiotemporal control of functional domains is needed so that the individual cell fate can be decided properly [84]. Thus, suitable hydrogels will act as bridges among cells as well as providing a “transportation system” within bioengineered tissues.

#### 3.1.2. Physicochemical Properties

Physicochemical properties are as significant as biological properties to biomaterials [85]. It has been recognized that physical parameters are important determinants for cell growth and phenotype regulation [85]. For instance, Jeremy Bomo et al. demonstrated that the proliferation rates of normal and transformed hepatocytes are strongly induced by matrix with a higher stiffness [86]. Another study demonstrated that primary hepatocyte functions were preserved when cultured on matrix of normal liver stiffness (400–600 Pa) but significantly reduced when cultured on matrix with the stiffness of fibrotic liver (1.2–1.6 kPa) [87]. In order to form an efficient “transportation system”, hydrogels have to gain more applicable mechanical properties besides suitable stiffness [88] such as mechanical stress and strength [79], elasticity and swelling, viscosity [83,89], and porosity [90,91]. For instance, hepatic cells are deposited within liver tissue with the stiffness around 640 Pa [92]. The pore size and porosity of scaffolds play an important role in the diffusion of growth factors and induce vascularization thereby aiding maintenance of liver specific functions [77]. As hepatocytes consume 5- to 10-fold more oxygen compared to other cells [93,94], pore size is a crucial factor which controls the mass transport of oxygen and nutrients into the interior of the scaffold, thereby supporting cellular growth in the region [95]. Porous scaffolds with pore sizes ranging from 50 to 150 μm and high inter-pore connectivity are desirable for the culture of hepatocytes [96]. Compared to hepatocytes cultured in the control scaffolds with non-uniform distribution of pores, primary hepatocytes cultured in a porous scaffold, owning a high porosity of around 83% with interconnected pores (average pore diameter 40–70 μm), showed an increase in albumin secretion and urea synthesis [97]. 

Apart from cellular and external influences, mechanical properties will also be affected by the materials themselves. In the in vivo ECM, the mechanical properties are largely influenced by proteoglycans and fibrous proteins. In the in vitro imitated ECM or implanted hydrogels, the mechanical properties are often influenced by the type and density of crosslinks. As the mechanics of the hydrogels affect the cell behavior and cell fate, mechanically patterned hydrogels have been created through local light exposure. Other influence factors include controllable variables such as concentration, polymer length and temperature [98]. On the other hand, natural ECMs have mechanical properties in a dynamic manner. Thus, hydrogel systems are designed with reversible mechanical properties to provide cells with optimal microenvironment in a spatiotemporal manner.

### 3.2. Categories of Hydrogels

Hydrogels could be distinguished with various parameters such as the preparation method, the overall charge, and the mechanical and structural characteristics. Here, the hydrogels are divided into two categories according to their origins: natural or synthetic.

#### 3.2.1. Natural Hydrogels

Natural hydrogels originate from organisms and have natural advantages to mimic the ECM better when compared to synthetic hydrogels. Generally, natural hydrogels function well for common uses such as cell culture, drug delivery, and tissue engineering. Several natural hydrogels have been used for LTE, including collagen, gelatin, hyaluronan, fibrin, alginate, chitosan, polyhydroxyalkanoates, cellulose, and agarose. Here we specify which natural hydrogels have been used (Table 1) and which main advantages and/or disadvantages exist for LTE.

Collagen is a significant constituent of the natural ECM and it consists of at least 19 subtypes that provide various functions. Collagen is naturally degraded by metalloproteases, specifically collagenase and serine proteases [8]. As a major determinant of the architecture and tensile strength of many tissues, collagen participates in numerous physiologically important interactions and was made into scaffolds, which have been used in a variety of applications due to a number of useful properties such as hemostatic, low antigenicity, and appropriate mechanical properties [103]. Collagen and glycosaminoglycans compose a considerable portion of the ECM to ensure the mechanical integrity of hepatocytes and are responsible for providing bioactive molecular signals to cells [112]. Platelet deposition and hepatocyte culture experiments showed that a new collagen/chitosan hydrogel had excellent blood and cell compatibility, which suggests that this hydrogel is a promising implantable candidate for LTE [79]. Andrea et al. optimized the collagen type I-hyaluronan hybrid hydrogel for liver microenvironments, which was employed to bioprint 3D liver tissue constructs containing primary human hepatocytes and liver stellate cells [113]. Similarly, collagen has been incorporated with other materials such as chitosan and heparin in order to recapitulate liver functions [80,114,115].

Gelatin is a protein produced by partial denaturalization or hydrolytic degradation of collagen and has a sol-gel transition temperature around 30 °C [104]. Due to its natural origin, gelatin possesses biological activities and has a high ability to form strong hydrogels and transparent films that are easily designed as insoluble hydrophilic polymers. Gelatin induced essential cellular functions, such as migration, proliferation and differentiation through integrin-mediated cell adhesion and cell-mediated enzymatic degradation [105]. Using rapid prototyping technology, hepatocytes were laminated into gelatin hydrogels for more than 30 layers, remained viable, and performed biological functions in the construct for more than two months [116]. More interestingly, a heparin–gelatin mixture was used to coat the vasculature within decellularized livers to reconstruct a patent vascular tree by seeding endothelial cells [117]. To make a whole bioengineered liver, gelatin was incorporated with polyurethane to generate a hydrogel with controlled pore size and interconnectivity for LTE [118].

Hyaluronan (hyaluronic acid, HA) is a non-sulphated glycosaminoglycan consisting of alternating units of D-glucuronic acid and D-N-acetylglucosamine, which are linked via beta-1,4 and beta-1,3 glycosidic bonds [99]. As one of the major components of ECM, HA is naturally degraded by hyaluronidase allowing cells in the body to regulate the clearance of the material in a localized manner. Unmodified HA binds to water and promotes swelling of the matrix and additionally can inhibit cell-cell adhesion by forming a porous coat around cells [119]. HA can also provide signals to enhance cell attachment and migration once modified with appropriate cell-adhesive proteins and peptides [104,120]. Therefore, HA has been used extensively for LTE applications. HA hydrogels used to be identified as the only culture condition that facilitated survival, proliferation and maintenance of hepatoblasts and could support human liver cells, including several subpopulations of hepatic progenitors [121]. Recently, Jonas et al. successfully cultured hepatocytes in a liver-on-a-chip setup by using a modular hyaluronan-PEG based 3D hydrogel modified with RGD peptides [122].

Fibrin can be isolated autologously from patients and fabricated into hydrogel scaffolds. Actually, fibrin was first noted to have a hemostatic effect on wounds and was subsequently applied to cerebral hemorrhage. With refinements adding to the strength, efficacy and safety, fibrin glues have become a more popular tool in the application of tissue-engineered skin replacements [109]. As fibrin can achieve high seeding efficiency and uniform cell distribution [110], fibrin hydrogels have also been utilized for LTE. Helge et al. evaluated a fibrin-based hydrogel and found it suitable for the stimulation of hepatocytes and it appeared to support engraftment and specific differentiation of viable hepatocytes [123]. Fibrin hydrogels together with PLGA and hepatocytes were assembled to an implantable liver tissue, along with a hierarchical vascular network [124]. Most recently, a fibrin hydrogel was successfully utilized for the ectopic expansion of engineered human liver tissue using mature cell populations [125].

Alginate is a polymer consisting of beta-D-mannuronic acid (M) and its alpha-L-glucuronic acid (G), and it is commonly found in the cell wall of brown seaweed and produced extracellularly by some bacteria [101,104]. As an anionic polysaccharide, alginate can easily create hydrogels in the presence of divalent cations and can mimic the ECM well, which makes it popular for LTE. One of the challenges in fabricating liver in vitro is the inability to culture hepatocytes. Using alginate-based scaffolds, hepatocytes were successfully cultured for two weeks while maintaining the hepatocyte phenotype [126]. Hence, scaffolds fabricated by 3D printing hold new promise in creating functional liver tissues [127]. Recently, an injectable hydrogel made from glycyrrhizin (GL), alginate (Alg), and calcium (Ca) was designed for application in LTE, and the GL–Alg–Ca hydrogel could maintain proliferation and liver specific functions of a hepatic cell line [128].

Chitosan is derived from the deacetylation of chitin, which is a linear polysaccharide consisting of beta-1,4 linked N-acetylglucosamine units. Chitin is the most abundant natural biopolymer besides cellulose and has highly hydrophobic and electric properties. Different from chitin, chitosan is a soluble polymer with high biofunctionality and better adsorption. Chitosan is capable of cell adherence and proliferation, and taking its ability to form highly porous scaffolds and antibacterial properties into consideration, chitosan is a promising choice for LTE. Pure chitosan-based microfibers were prepared to support self-aggregation of liver cells into spheroids, showing improved liver specific functions [129]. He et al. made use of well-organized microstructures for hepatic tissue engineering with chitosan-gelatin hybrid scaffolds [130]. Furthermore, with the fibronectin coating on the surface, the chitosan nanofibers exhibited a significantly enhanced cell attachment and the hepatocytes in co-cultures formed colonies and maintained their morphologies and functions for prolonged periods of time [131].

Polyhydroxyalkanoates (PHAs) are a group of aliphatic polyesters synthesized by bacteria to store intracellular carbon and energy, including more than 150 identified monomers [29]. Various monomers provide a broad range of properties to engineer multifunctional constructs that have poor stiffness and slow degradation rate. Su et al. [132] developed scaffolds for LTE using poly(3-hydroxybutyrate-co-3-hydroxyvalerate-co-3-hydroxyhexanoate) (PHBVHHx). The matrices derived were loaded with human umbilical cord multipotent stromal cells (MSCs) and hepatocyte-like cells, and after 28 days the tissue generated looked very similar to the native organ. A study reported the recovery of injured mouse liver when a PHBVHHx scaffold loaded with human umbilical cord Wharton’s jelly (WJ) MSCs was transplanted [133]. Chemically modified PHAs also find use as films, pins, sutures, screws, and scaffolds for repairing skin, cartilage and LTE [111].

Cellulose: In contrast with most other biopolymers, gelation of various cellulose derivatives including MC and hydroxypropylmethylcellulose (HPMC) occurs upon heating. Cellulose is often combined with proteins (e.g., gelatin), polysaccharides (e.g., chitosan), or both. Other cellulose derivatives have been reviewed by Vlierberghe, et al. [104]. Cellulose nanofibrils, which are fibrils in the nanometer range, show general properties of cellulose: hydrophilicity and broad chemical modification capacity combined with properties specific for nanoscale materials due to their high surface area. With good mechanical properties and biocompatibility, cellulose nanofibrils are attractive for biomedical applications [32,134]. Nanofibrillar cellulose hydrogel was shown to promote three-dimensional liver cell culture [82]. A hydrogel composed of alginate and cellulose nanocrystal was suitable for bioprinting of liver-mimetic honeycomb 3D structures [135]. Wood-derived nanofibrillar cellulose (NFC) has been incorporated with hyaluronan-gelatin (HG) to form hydrogels for the differentiation of liver progenitors, and undifferentiated progenitor cells in NFC-HG hydrogels formed 3D multicellular spheroids with apicobasal polarity and functional bile canaliculi-like structures, structural hallmarks of the liver tissue [136].

Agarose is a linear polysaccharide formed by the disaccharide of beta-D-galactose and 3,6-anhydro-a-L-galactopyranose. Agarose is extracted from seaweed and can be dissolved in hot water. It forms a gel upon cooling due to the formation of double helices and their subsequent aggregation. The thermo-reversible gelation process depends on the type of agarose or methoxy content [97]. Agarose gels have adjustable pore sizes and are physicochemically strong, which enables high diffusion rates. Primary hepatocytes could proliferate in vitro in an agarose-chitosan scaffold, with suitable physicochemical properties and hepatic cell compatibility, and showed an increase in cellular metabolic activity. Hepatic functions like albumin secretion and urea synthesis were improved for primary hepatocytes in the 3D scaffold compared to controls [97].

#### 3.2.2. Synthetic Hydrogels

Synthetic hydrogels are artificial hydrogels with a defined composition and structure. Compared to biological hydrogels, synthetic hydrogels are less complex and have stronger mechanical structure, less animal origination, are well controlled, commercially friendly, and relatively easier to be FDA-approved, which makes them more and more popular. Several synthetic materials utilized to make hydrogels are introduced in the following section and Table 2.

Poly(ethylene glycol) (PEG) is a polyether compound which is water-soluble, amphiphilic, transparent, colorless, liquid, and viscous. Various modifications have been applied to PEG to enhance the mechanical properties for 3D printing, to contribute to high elasticity, or to increase hydrophilicity which could tune the degradation rate [78]. PEG derivatives were used as crosslinkers to develop bioartificial vessel-like grafts [146]. Nowadays, it has become a frequently employed strategy to increase protein solubility and stability to reduce immunogenicity and to alter circulation half-life [139]. For LTE, PEG hydrogels are widely used for encapsulation, and was shown to provide a biocompatible matrix that allows the majority of encapsulated primary hepatocytes to survive [147]. The survival and function of PEG hydrogel-encapsulated hepatic cells have been improved by modifications in polymer chain length and the conjugation of bioactive factors [8]. Moreover, hepatic cells have been encapsulated well into the photopolymerized PEG hydrogel through which complex architecture constructs were assembled [148]. The undegradable PEG hydrogel was applied for the encapsulation of co-cultured hepatocytes, preventing aggregation and overgrowth, and enabling formation of microtissues with stable hepatic function [149]. Recently, PEG was fabricated into 3D hexagonally arrayed lobular human liver tissues and the hydrogel enabled primary human fetal liver cells to self-assemble into a 3D configuration and preserved advanced hepatic functions for at least five months [150].

Polyisocyanopeptide (PIC) is an innovative and fully synthetic polymer, capable of mimicking characteristics of the natural ECM [139]. PIC exhibits thermo-reversible behavior due to the hydrophobic interactions of the oligoglycol substituent present along its backbone, with a steep increase of the storage modulus (G’) above 18 °C. As a water-soluble synthetic polymer, PIC mimics natural protein-based filaments. Its thermoreversible gelation property and cytocompatibility make PIC an ideal candidate for bioprinting technology [151]. The unique semiflexible properties combined with a length of several hundred nanometers have recently made it particularly attractive for LTE [139]. 

Poly(vinyl alcohol) (PVA) is prepared in two steps due to the unstable form of vinyl alcohol as monomeric units. By controlling the hydrolysis step, different grades of PVA polymer can be prepared, which finally affects the behavior of the polymer material, solubility, crystallinity, and chemical properties [152]. PVA-based hydrogels have been applied to many kinds of tissues, such as skin, bone, cartilage, vascular- and cardiac-tissue, human prostate and artificial cornea. Due to its favorable properties and easy manipulation, PVA-based hydrogels have been recognized as promising biomaterials and are suitable candidates for LTE. To overcome disadvantages such as poor cell-adhesion, they still need further modifications for targeted applications [36]. Shan et al. developed a method to prepare transparent PVA hydrogels by varying the freeze/thaw cycles and the PVA hydrogels exhibited similar mechanical properties and morphological characteristics to that of a porcine liver, a reference material for human soft tissue [137]. PVA/gelatin hydrogels were proposed as a 3D microenvironment for liver cells to form an in vitro hepatocellular carcinoma model [153].

Poly(lactide-co-glycolide) acid (PLGA) is an FDA approved biodegradable material and has been studied widely both in vivo and in vitro. Previous studies have shown poor load bearing properties [141]. Due to its biocompatibility and controllable biodegradability, PLGA microspheres have been utilized as scaffolds containing cells to enhance the vascularization of engineered tissues. Besides, PLGA is also attractive for its property to be degraded by hydrolysis to lactic acid and glycolic acid [142]. Therefore, PLGA hydrogels have been used frequently for LTE. More than 20 years ago, PLGA was fabricated into scaffolds for LTE and seeded with hepatocytes and non-parenchymal cells from rats [154]. When cultured together with biodegradable PLGA membranes, the cells in the 3D stacked structures recovered polarity and exhibited improved liver-specific functions as compared with cells in a monolayer [155]. Moreover, the transdifferentiation rates of bone marrow mesenchymal stem cells (BMSCs) to mature hepatocytes were improved by collagen-coated PLGA [84]. Recently, PLGA polymer has been utilized to fabricate an absorbable vascular anastomosis device and the device was tested in pig liver transplantation experiments, where it was successfully absorbed within four months [83].

Poly(glycolide) acid (PGA) is the simplest linear aliphatic polyester and was used to develop the first totally synthetic absorbable suture. With a high degree of crystallization and high melting point, PGA is not soluble in most organic solvents except for the highly fluorinated ones. As an absorbable material, its thermal stability is good. Unfortunately, PGA tends to lose its mechanical strength rapidly due to the hydrophilic nature. Sutures of PGA will lose around 50% of their strength after two weeks and 100% at four weeks, and will get completely absorbed in 4–6 months [143]. With this property, a PGA felt was incorporated with fibrin sealant for prevention of bile leakage after liver resection [156].

Poly(lactide) (PLA) lactide is the cyclic dimer of lactic acid with two optical isomers. L-lactide is the naturally occurring isomer and DL-lactide is the synthetic blend of D- and L-lactide. The polymerization of lactide is similar to that of PGA. With a pendant methyl group on the alpha carbon, PLAs are quite different in chemical, physical and mechanical properties when compared to PGA, even though their structures are similar, and PLA is more frequently utilized in LTE. Rat hepatocytes cocultured with primary rat hepatic stellate cells on the PLA hydrogels have been shown to maintain hepato-specific functions for more than two months [157]. The biodegradable copolymer poly(lactic acid-co-lysine) (PLAL) contributed to hepatocyte engraftment, function and expansion [158]. Type I collagen coated electrospun poly(L-lactic acid) (PLLA) nanofibers with random and aligned orientation were evaluated for hepatocyte adhesion and proliferation [159]. PLLA and gelatin were used to induce hepatic differentiation of MSCs in the form of electrospun nanofiber scaffolds and the microporous scaffolds controlled the migration of hepatic stellate cells through pore size [9].

Poly(e-caprolactone) (PCL) is an aliphatic polyester with a glass transition temperature at −60 °C. The ring-opening polymerization of e-caprolactone yields a semi crystalline polymer and gives softness and flexibility at near body temperature. This polymer has been regarded as tissue-compatible and used as a biodegradable suture in Europe. Furthermore, the very low degradation rate makes it suitable for long-term implants or for drug delivery systems [148]. PCL combinations with a variety of natural polymers were reported for LTE [145]. PCL has been used to enhance mechanical properties and could be bioprinted together with hepatocytes, endothelial cells and fibroblasts, which maintained hepatocyte functions and facilitated the formation of vascular networks [160]. Rhiannon et al. developed hybrid PCL-ECM scaffolds for LTE, which maintained hepatocyte growth and function [161]. In addition, PCL nanofiber scaffolds supported the in vitro differentiation of human somatic stem cells into hepatocytes [162]. Besides hepatocytes, PCL/chitosan electrospun nanofibers were evaluated to be competent for the culture of mouse hepatic cells, indicating that PCL/chitosan hydrogels would be excellent for LTE [163].

Poly(acrylic acid) (PAA) and its derivatives are among the most intensively studied synthetic materials for biomedical applications. Several attempts have been made for their application in LTE. When grafted within PAA, the growth kinetics of adhesion patch at primary hepatocyte cell substrate interface was changed [164]. Amol et al. [165] conjugated PAA and polyethyleneimine (PEI) with elastin-like polypeptides (ELPs) and found that the conjugates influenced the morphology, aggregation and differentiation function of primary rat hepatocytes.

### 3.3. Progress in Hydrogel Techniques

Great progress has been achieved in hydrogel techniques to provide as many cues as possible for mimicking the ECM. As mentioned above, various design strategies to overcome the shortcomings of individual biomaterials were developed and many different hydrogels that successfully mimic the complexity of natural ECMs have been created.

The aim of different design strategies is simply to make the best use of ideal characteristics of various biomaterials and recapitulate as many ECM functions as possible. The principle to design those hydrogels is based on the properties required by the liver. Up till recently, properties such as biocompatibility, biodegradability, adhesive property, thermal-responsiveness and purposed stiffness and swelling have come into being via elaborate designs.

Multidisciplinary design optimization has been tried to devise ideal hydrogels (Table 3). Several aspects have been studied in great detail, such as gel formation dynamics, crosslinking modes, and mechanical and degradable material linkages. These properties are linked to the intrinsic properties of the main chain polymer and the crosslinking characteristics (amount, type, and size of crosslinking molecules) [8]. The most common types of crosslink include: covalent, physical, dynamic covalent, hydrogen bonding, affinity bonding, hydrophobic interaction, and chain entanglement. Compared to chemical means, which may be toxic and could affect the nature of substances, physical mechanisms are safer. Different from conventional chemical or physical methods, photopolymerization seems more promising and is attracting more attention. 

Methods mentioned above provide various possibilities for hydrogel design [140]. Hydrogel compositions can be reinforced by polymers or other hydrogels. To promote cell adhesion, peptides and fragments are used. Polymeric hydrogel adhesives could be synthesized by physical or chemical gelation or by the combination of both. Different materials (polysaccharide-/protein-/or synthetic polymer-) based hydrogel adhesives own quite different characteristics, signaling properties included. For example, the design of galactose-carrying hydrogels as ECMs can guide hepatocyte adhesion and enhance cell functions [166]. Another highly studied property is the degradability of hydrogels. At present, hydrolysis and enzymatic methods are still the main strategies for hydrogel degradation.

With the combination of multiple design strategies, hydrogels tend to gain more comprehensive properties and methods to characterize properties of hydrogels have also been increasing. Up till now, the most frequently characterized properties are gelation time, gel fraction, swelling degree, structural parameters, water vapor transmission rate, and mechanical properties. Smart hydrogels with high tunability of stiffness can be designed with various modifications, which enable hydrogels to be pH-/ thermo-/ photo-/ redox-, or mechano-responsive [92]. 

## 4. Conclusions

**Figure 2:**
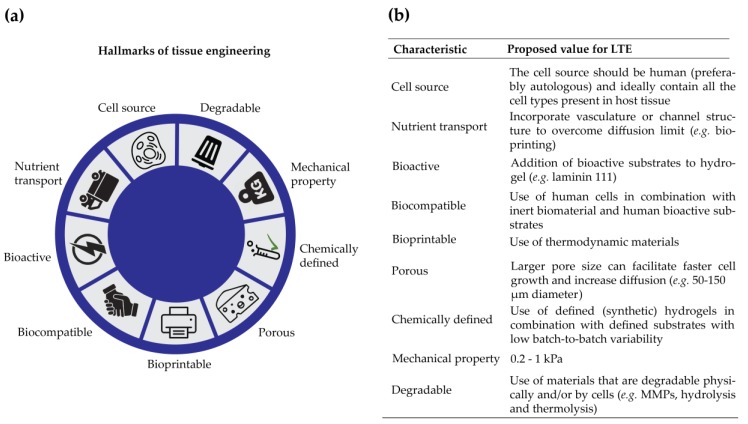
Hydrogels for liver tissue engineering (LTE). (**a**) Hallmarks of tissue engineering; (**b**) Proposed value of characteristics specific for LTE.

Taking the biological and physicochemical properties into consideration, the characteristics that are significant for LTE are summarized (Figure 2a) and some specific properties are suggested (Figure 2b), which may facilitate the choice for a specific hydrogel to mimic ECM for LTE.

In view of the biological origin of natural materials, the majority is biocompatible, biodegradable, and abundantly available. As most of these natural materials are present in ECM, cells have a good compatibility and growth response. Being more bioactive compared to synthetic hydrogels, natural hydrogels have a longer history of research as well as more utilization in TERM, especially since several of them have been FDA-approved. However, every coin has two sides, and this is also true with regard to natural hydrogels. Compared with synthetic materials, natural hydrogels have several shortcomings, such as mechanical weakness, batch-to-batch variability, and the fact that some are animal-derived, which implies ethical issues and restricts the utility for clinical applications. Obviously these drawbacks do not, or to a lesser degree, account for synthetic hydrogels.

Synthetic hydrogels are either modified from natural materials or completely synthetic, and based on the type of material, synthetic hydrogels offer choices to be degradable or nondegradable. Compared to natural hydrogels, synthetic hydrogels are relatively less immunogenic, quality-reproducible, mechanically stronger, and easily modifiable [158]. Strong mechanical properties and various modifications have increased the popularity of synthetic hydrogels in TERM. However, synthetic hydrogels are still far from perfect for LTE, and the most significant weakness is that many of them are less bioactive and lack viscoelasticity. 

As neither natural-nor synthetic-hydrogels alone are suitable for LTE, the combination of different hydrogels, with different origins or various modifications, has been applied in LTE, and great progress has been achieved in the past decades. As an example, the overall performance of the liver cell-loaded PCL scaffolds was remarkably improved by avidin–biotin binding-based cell seeding [190]. In addition, it has been shown that presence of poly(3,4-ethylenedioxythiophene) (PEDOT) as a conducting polymer in the scaffolds, with the combination of gelatin/ chitosan/ hyaluronan, enhanced hepatocyte cell viability, attachment and proliferation [191].

## 5. Discussion

Constructing a physiologically relevant bioengineered liver is of great interest as an in vitro model for fundamental and applied research such as disease pathogenesis, drug metabolism, and toxicological studies. Moreover, building physiologically relevant models now for in vitro studies will at the same time enhance our knowledge and progress towards LTE for clinical applications such as OLT in the future. Hydrogels are one of the most vital ingredients besides cells for bioengineered livers. Although great progress has been made in the past decades, there are still several major issues to be taken into consideration.

First of all, multiple liver cell types should be included to make a more physiologically relevant liver and characteristics required for hydrogels may vary among different cell types. In addition, bioengineering technologies have to allow the spatial orientation of these hydrogels in order to be planted at different positions to form microstructures. For example, viability and hepatic cell function were improved in micropatterned constructs as compared to unpatterned controls, demonstrating the importance of recreating the native microarchitectural features [91].

Secondly, there is a necessity to gain comprehensive understanding of liver ECMs. Liver ECM takes up 16–22% of the total liver volume [68,192,193], and is composed of various cues that can be divided into three categories [85]: supportive structure made from insoluble hydrated macromolecules (e.g., fibrillar proteins, proteoglycans, or polymer chains), soluble molecules (e.g., growth factors or cytokines), and noncellular factors (e.g., pH, temperature, charge). All those ECM effectors are possible determinants for the cell fate, interaction among cells, and the structure and function of tissues or organs. Similarly, liver cells can respond differently to various ECM components. Moreover, the ECM composition also varies in different parts of the liver (Figure 1c), which makes mimicking ECM for LTE more challenging. Therefore, several groups tried to use decellularized liver ECM as bioink for 3D cell-printing based LTE [194,195]. Nevertheless, the undefined chemical components of decellularized liver ECM will also restrict future applications in clinical treatment. Therefore, chemically defined hydrogels are still more promising for LTE. As more cues from liver specific ECM will be discovered, especially for the ECM within the space of Disse and the sinusoidal lumen, synthetic hydrogels will be able to mimic the in vivo microenvironment in much more detail. 

Selection and design of hydrogels has to be carefully considered, and might differ depending on different applications [196]. To closer mimic the natural liver ECM, more details need to be included, which sets various strict requirements for hydrogels (Figure 2a). These requirements include: gel formation dynamics, crosslinking modes, biological and physicochemical properties, and degradable linkages. Importantly, the studies that are reviewed here and summarized in Table 2 did not only use different materials to mimic the ECM but also applied several different cell sources, cross-linking methods etc. This makes it difficult to directly compare the studies to each other, and to translate the outcome from one study to another. Nevertheless, some general conclusions on the requirements of hydrogels for LTE can be drawn, which are summarized in Figure 2b. For instance, the most dynamic effects of ECM stiffness on primary hepatocyte morphology and function were in the relatively narrow range between 150 Pa, the stiffness of normal liver, and 1 kPa, the lower threshold of fibrotic liver stiffness [87]. Primary hepatocytes demonstrated high viability and proliferation when seeded on 3D-printed gelatin scaffolds with precisely controlled pore geometry, and a physiologically mimetic 3D environment was proposed to be necessary to induce both expression and function of cultured hepatocytes [197]. Apart from those theoretical demands, several practical requests should also be kept in mind, especially for clinical applications. For instance, the hydrogels should be nonimmunogenic, easy to sterilize, and should enable engraftment post-implantation, being physically tunable to the in vivo microenvironment and the vascularization that has to be achieved within two days so that cells can survive and function.

In addition, related technologies have to keep up with the development of advanced hydrogels and their exquisitely designed characteristics, such as robust analysis technologies for local measurements of mechanical properties, and nanotechnology and bioprinting for promoting LTE.

## Figures and Tables

**Table 1:** Characteristics and applications of natural materials.

Natural Materials	Major Concerned Properties						Applications	References
	Biocompatible	Biodegradable	Hydrophilic	Thermal-Responsible	FDA	Other Characteristics		
**HA**	Yes	Yes, hyaluronidases	Yes	Yes	Approved	Nonimmunogenic, non-adhesive, good swelling and creep compliance properties, photopolymerizable, promote cell mortality and proliferation, reduces long-term inflammation, hepatic elimination	Tissue engineering, wound healing, angiogenesis, etc.	[99,100]
**Alginate**	Yes	Yes, controllable	Yes	Yes	Approved	Nonimmunogenic, bioactive, ease of gelation, suitable for in situ injection; poor elasticity, poor cell adhesion, mechanical weakness, difficulties in handling and sterilization	Tissue engineering and regeneration, as model ECMs, drug delivery	[101,102]
**Collagen**	Yes	Yes	Yes	Yes	Approved	Good permeability, low immunogenicity, poor mechanical properties	Tissue engineering (including cartilage, ligament, vessel etc.)	[34,103]
**Gelatin**	Yes	Yes, very fast	Yes	Yes	Approved	Ease of manipulation, high mechanical properties, easy to form films and matrix hydrogels, very viscous polymer, low thermal stability, low cost	Tissue engineering, drug discovery	[104,105]
**Cellulose**	Yes	Yes	Yes	Yes	Approved	Noncytotoxic, good thermal and mechanical properties, hydrogels with a high water content	Various derivatives in biomedical field	[32]
**Matrigel**	Yes	Yes	Yes	Yes	No	Bioactive, mechanical weakness, batch-to-batch variation, undefined composition	3D models, cell culture, mimic ECMs	[106,107,108]
**Chitosan**	Yes	Yes, lysozyme	Yes	Yes	Approved	Nonimmunogenic, good host response, high antimicrobial activity, very viscous polymer solution and pH-responsive, sufficient mechanical properties	Tissue engineering, e.g., liver, bone, skin, vessels	[31,89]
**Agarose**	Yes	Yes	Yes	Yes	No	High mechanical strength, ability to maintain the cellular phenotype	Mimics the microenvironment for hepatocytes	[97]
**Fibrin**	Yes	Yes	No	Yes	Approved	Easily autologous isolation, uniform cell distribution, limited mechanical strength, inflammatory response	Tissue engineering scaffolds, blood blotting, fertility preservation	[33,109,110]
**PHAs**	Yes	Yes	No	Yes	No	Non-toxic, piezoelectric properties, brittleness, tendency to crystallize	Tissue engineering including LTE, drug carrier, would healing	[29,111]

**Abbreviations**: Hyaluronic acid, HA; polyhydroxyalkanoates, PHAs; liver tissue engineering, LTE.

**Table 2:** Characteristics and applications of synthetic materials.

Synthetic Materials	Major Concerned Properties						Applications	References
	Biocompatible	Biodegradable	Hydrophilic	Thermal-Responsive	FDA	Other Characteristics		
**PAA**	Yes	No	Yes	No	Approved	Various derivatives, excellent optical transparency and stability in water	Degradable or thermal-responsive derivatives for drug delivery	[31]
**PVA**	Yes	No	Yes	Yes	Approved	Inefficient elasticity, stiff membrane, lack of cell adhesion, ease of fabrication and sterilization	Tissue engineering, both medical and nonmedical devices	[36,137]
**PIC**	Yes	No	Yes	Yes	No	Semiflexible properties, strain stiffening	Tissue engineering and cancer immunotherapy	[138,139]
**PEG**	Yes	No	Yes	No	Approved	Hydrolytically bioactive, photocrosslinkable, easily modifiable	Widely used in for chemical, biological, and commercial purposes, and also in tissue engineering	[31,140]
**PLGA**	Yes	Yes, controlled	No	Yes	Approved	Poor load-bearing properties, hydrolytically unstable; good cell adhesion and proliferation	Medical devices, drug delivery, fabrication of tissue engineering matrices, suture reinforcements	[141,142]
**PGA**	Yes	Yes	Yes	No	Approved	Highly crystalline, high melting point, lacks elasticity, not soluble in most organic solvents, tends to lose mechanical strength	Absorbable sutures, orthopedic devices, scaffolding matrices for tissue regeneration	[81,143]
**PLA**	Yes	Yes, slow	No	No	Approved	Lacks elasticity, high tensile strength, hydrolytically unstable	Orthopedic fixation devices	[143,144]
**PCL**	Yes, less	Yes. Low rate	No	Yes	Approved	Limited elasticity, tunable mechanical properties	Tissue engineering, long-term drug/vaccine delivery vehicle	[35,145]

**Abbreviations**: poly(ethylene glycol), PEG; poly(lactide-co-glycolide) acid, PLGA; polyisocyano peptide, PIC; Poly(vinyl alcohol), PVA; poly(N-vinylpyrrolid), PVP; poly(propylene furmarate-co-ethylene glycol), P(PF-co-EG); poly(2-hydroxyethylmethacrylate), HEMA; poly(acrylic acid), PAA; polyglycolic acid, PGA; polylactic acid, PLA; poly-e-caprolactone, PCL; poly(N-isopropylacrylamide), PNIPAAm.

**Table 3:** Hydrogels designed for liver tissue engineering.

Composition	Cell Source	Crosslinking Method	Output	Reference
Collagen, chitosan	Platelet and rat hepatocyte; rat hepatocyte	Chemical crosslinking; noncovalently linked	The matrix has excellent blood and cell compatibility; hepatocytes exhibited relatively high glutamate-oxaloacetate transaminase and glucose secretion functions	[79,114]
Collagen, chitosan, heparin	Platelet and rat hepatocyte	Chemical and physical crosslinking	Improved the blood compatability and maintained hepatocyte viability and function; exhibited high urea and triglyceride secretion functions	[80,113,115]
Collagen I, HA	Primary human hepatocytes and liver stellate cells	Physical crosslinking, UV crosslinker	Bioprinted 3D liver tissue constructs maintained liver functions including urea and albumin production	[113]
Gelatin, chitosan	Human HepG2; primary rat hepatocyte	Crosslinked with 1% genipin; crosslinked by glutaraldehyde solution	Cells cultured in 3D scaffolds preformed better on the structural characteristics, cell viability, growth and liver specific functions; supply living cells with nutrients and allow removing the cell metabolite; hepatocytes perform better in the well-defined scaffold	[167,168,169]
Gelatin, silk fibroin (SF)	Human normal hepatic QZG cell line	Use of glutaraldehyde solution to produce cross-linked gelatin solution and then mix with SF	Achieved better biocompatibility, controlled degradation, and good for the attachment and proliferation of cells	[170]
Gelatin	Primary rat hepatocytes	Gelatin is dissolved in hot NaCl and Tris-HCl	Rapid prototypedg hepatocytes remained viable and performed biological functions for more than 2 months	[116]
Gelatin, heparin	Human endothelial cells and HepG2 cells	Physical mixture	Helped cells to reconstruct a patent vascular tree within the decellularized porcine liver scaffold	[117]
Gelatin, polyurethane	Hepatocyte	Cross-linked with glutaraldehyde, enhanced by the addition of lysine	Generation of a hydrogel with controlled pore size and interconnectivity	[118]
GelMA	Human HepG2/C3A cells	Photocrosslinked	Bioprinted liver spheroids exhibited long-term functionality	[112]
HA, PEG	Human HepG2 cells, hiPS-HEPs	Bioorthogonal SPAAC crosslinked, modified with cyclic RGD peptides	hiPS-HEPs migrated and grew in 3D and showed an increased viability and higher albumin production compared to ctrols	[122]
HA, moieties; collagen III, laminin	Primary rat hepatocytes; hHpSCs	Galactose moieties were covalently coupled with HA through ethylenediamine; the is initiated by a PEGDA cross-linker	Formation of cellular aggregates with enhanced liver specific metabolic activities and improved cell density; permissive for survival and phenotypic stability of human hepatic stem cells and hepatoblasts	[171,172]
Fibrin	Rat hepatocyte; human hepatocytes, dermal fibroblasts, and UVECs	Human fibrinogen was applied with the thrombin solution to make the fibrin matrix	Supported engraftment and specific differentiation of viable hepatocytes; stimulated hepatocytes for the ectopic expansion of engineered human liver tissue seeds; in vitro-generated liver tissues can expand and function in vivo	[110,123,125]
Fibrin, PLGA	Rat hepatocytes and ADSCs	Formed by the pollymerization of fibrinogen acted by protease thrombin	Assembled to be an implantable endothelialized liver tissue, along with a hierarchical vascular network	[124]
Alginate	Mouse primary hepatocytes; HepG2 cells	Freezedry technique; crosslinked in CaCl2 solution	Maintained hepatocyte genotype, produced hepatic-specific proteins for two weeks; liver spheroids displayed an enhanced cell proliferation; importance of cell density within weakly adhesive alginate scaffolds; a cold reduction in temperature display an enhanced cell proliferation	[101,126,127,173,174]
Alginate, galactosylated chitosan	Primary hepatocytes	Calcium crosslinked; lyophilization	Enhanced hepatocyte aggregation; improved cell attachment and viability	[175,176]
Alginate (Alg), glycyrrhizin (GL), calcium (Ca)	HepG2 cells	Calcium crosslinked equal volume mixture of GL, nano-CaCO3 and Alg	GL–Alg–Ca hydrogel was homogenous complex with stable structure and well viscoelasticity, and cells showed good biocompatibility, and maintained the viability, proliferation and liver function	[128]
Chitosan	HepG2 cells	The microfluidic fabrication process for pure chitosan microfibers	HepG2 cells were self-aggregated with a spheroid shape, showing a higher liver specific function (albumin secretion and urea synthesis).	[129]
Lactose-modified chitosan (Lact-CTS)	Normal liver cell	Coupling of lactose with chitosan was carried out by the reducing agent, addition of NaBH4	Lact-CTS with 48.62% of galactose moieties could facilitate the cell attachment and possess great biocompatibility and mechanical stability	[177]
Chitosan, gelatin	Hepatoytes	Crosslinked by glutaraldehyde solution	Scaffold produced with predefined multilevel internal architectures (a flow-channel network and hepatic chambers) and improved hepatocytes performance greatly in comparison with a porous scaffold	[130]
Silk fibroin/chitosan (SFCS)	HepG2 cells	Freezing and lyophilization	Provided a matrix with homogeneous porous structure, controllable pore size and mechanical properties	[178,179]
Chitosan nanofibers, fibronectin	Primary rat hepatocytes, endothelial cells	Fabricated by the electrospinning technique	Enhanced cell attachment and maintained their morphologies and functions	[131]
PHBVHHx	UC-MSCs, hepatocyte cells	Solid–liquid phase separation method to form scaffolds	Injured mice liver were recovered; generated tissue looked similar to the organ	[132,133]
Native nanofibrillar cellulose (NFC)	Human hepatic cell lines HepaRG and HepG2	Physically crosslinked	Provided mechanical support for cell growth and differentiation, and induced spheroid formation of HepaRG and HepG2 cells.	[82]
Cellulose nanocrystral (CNCs), alginate	Human hepatoma cells, fibroblasts	Crosslinked with CaCl2	The bioink formulation was suitable to print a liver mimetic honeycomb 3D structure containing fibroblast and heptatoma cells	[135]
Nanofibrillar cellulose, HA-gelatin	Human HepaRG liver progenitor cells,	HG hydrogel based on thiol-modified HA, thiol-modified gelatin and crosslinker PEGDA	Induced apicobasal polarity and functional bile canaliculi-like structures, expediting the hepatic differentiation of HepaRG liver progenitor cells better than the standard 2D culture	[136]
Agarose, carbohydrate glass	Primary rat hepatocytes and fibroblasts	Chain entanglements, physical crosslinking	Primary hepatocytes and fibroblasts were cast	[180,181]
Agarose–chitosan (AG–CH)	Primary rat hepatocytes	Crosslinke by glutaraldehyde	The hepatic functions like albumin secretion and urea synthesis were established in the 3D scaffold	[97]
PEG, heparin	Primary rat hepatocytes, BMEL; cryopreserved primary human hepatocytes, induced pluripotent stem cells (iPSCs)	UV light polymerization; chemically crosslinked	Demonstrated the importance of cell–cell and cell–matrix interactions in BMEL cell and primary hepatocyte survival. Aggregation and encapsulation of iPS cells during their differentiation towards hepatocytes yielded microtissues that depicted stable albumin production on-chip and inducible CYP activity. The 3D in vitro liver model is capable of sustaining advanced human-specific liver functions	[147,148,149,182,183]
PEG, PLGA, liver-derived ECM (L-ECM), growth factors	Rat liver	Physical and thermal crosslinking	L-ECM and growth factors enhanced tissue penetration into intrahepatically implanted biodegradable scaffolds and induced cell proliferation in the parenchyma that surrounds these scaffolds in the normal liver	[184]
PEG-DA, PEGDAAm, MMP-sensitive	Primary human fetal liver cells, HUVECs and HepG2	Chemical crosslinking, photopolymerization	The 3D in vitro liver model is capable of sustaining advanced human-specific liver functions for at least 5 months in culture. Hepatic tissues survived and functioned for over 3 weeks after implantation	[150,185]
PIC, GRGDS peptide	Human dermal microvascular endothelial cells and fibroblasts	Polymerization of the corresponding monomers using a nickel perchlorate as a catalyst.	Supported pre-vascularization and the development of organotypic structures	[186]
PVA		Physical crosslinking by freeze–thaw cycless	Exhibited similar mechanical properties and morphological characteristics to porcine liver	[137]
PVA, gelatin	HUVECs and HepG2	Physically crosslinked by freeze–thaw cycles	Hydrogel particles with a well pronounced tendency towards association with hepatocytes and endothelial cells.	[152]
PLGA, AVAD; collagen-coated PLGA (C-PLGA); PLGA, gelatin	Pig liver transplantation; rat BMSCs; rat hepatocytes (HCs), nonparenchymal liver cells	Chemical and physical crosslinking	Demonstrated the feasibility of using AVADs in organ transplantation. Proved the superiority of the C-PLGA for hepatocytes differentiation. HCs cocultured with nonparenchymal cells can attach to and survive on the 3D polymer scaffolds. Cells recovered polarity and exhibited improved liver-specific function. Untreated PLGA performed best for supporting liver-specific functions. 3D printing and optimized parameters are applied for liver regeneration.	[83,84,154,155,187,188]
PGA, fibrin		Physical crosslinking	Effective in preventing biliary leakage	[156]
PLA	Rat hepatocytes, rat hepatic stellate cells	Dissolved in 2,2,2-trifluoroethanol	Encouraged the rapid self-organization of 3D spheroids and the spheroids formed exhibit hepatocyte-specific functionality	[157]
PLAL	Rat hepatocyte	Chemical crosslinking	Contributed to hepatocyte engraftment, function, and expansion	[158]
PLA, fibroin; collagen I; discrete aligned nanofibers	HepG2 cells; rat hepatocyte	Chemical and thermal crosslinking	Improved the cell growth, enhancing cells adhesion and proliferation. Hepatocyte aggregates formed on nanofibers displayed excellent cell retention, cell activity and stable functional expression	[144,159]
PLLA, gelatin, HGF	BMSCs	Electrospinning, physical and chemical crosslinking	Effectively guide hepatic commitment of patient derived BMSCs	[9,189]
PCL, collagen	Primary rat hepatocytes, HUVECs and human lung fibroblasts (HLFs)	Physical and thermal crosslinking, 3D printing	The vascular formation and functional abilities of HCs demonstrated that the heterotypic interaction among HCs and nonparenchymal cells increased the survivability and functionality of HCs	[160]
PCL, ECM	HepG2 hepatocytes	Electrospinning, physical and thermal crosslinking	Provided a viable, translatable platform for hepatocytes, supporting in vivo phenotype and function	[161]
PCL	Human USSCs, self-renewing pluripotent cells	Physical crosslinking, electrospinning	Differentiation of USSCs demonstrated that this culture system can potentially be used as an alternative to the ECM-based culture for relevant hepatocyte-based applications in LTE	[162]
PCL, chitosan	Epithelial liver mouse cells.	Physical crosslinking, electrospinning	The porosity and pore is suitable for epithelial liver mouse cells infiltration, attachment, and material exchange	[163]
PAA, PET, collagen	Primary hepatocytes	Chemical an physical crosslinking, UV light induced polymerization	The growth kinetics of adhesion patch at primary hepatocyte cell substrate interface is changed upon PAA grafting	[164]
PAA, PEI, ELPs	Primary rat hepatocytes,	Chemical an physical crosslinking	ELP–polyelectrolyte conjugates profoundly influenced the morphology, aggregation and differentiation function of primary rat hepatocytes	[165]

**Abbreviations:** hyaluronic acid, HA; polyhydroxyalkanoates, PHAs; poly(ethylene glycol), PEG; poly(lactide-co-glycolide) acid, PLGA; polyisocyano peptide, PIC; poly(vinyl alcohol), PVA; poly(N-vinylpyrrolid), PVP; poly(propylene furmarate-co-ethylene glycol), P(PF-co-EG); felatin methacryloyl, GelMA;poly(acrylic acid), PAA; poly(ethylene terephthalate), PET; polyethyleneimine, PEI; polyglycolic acid, PGA; polylactic acid, PLA; poly-e-caprolactone, PCL; poly(N-isopropylacrylamide), PNIPAAm; hepatoma cells, HepG2; strain-promoted alkyne-azide 1,3-dipolar cycloaddition, SPAAC; Arg-Gly-Asp, RGD; bone marrow mesenchymal stem cells, BMSCs; matrixmetalloproteinase sensitive peptide, MMP-sensitive; human iPSC derived hepatocytes, hiPS-HEPs; human hepatic stem cells, hHpSCs; (human) umbilical vein endothelial cells, (H)UVECs; adipose-derived stem cells, ADSCs; bipotential mouse embryonic liver cells, BMEL; absorbable vascular anastomotic device, AVAD; elastin-like polypeptides, ELPs; human cord blood-derived unrestricted somatic stem cells, USSCs.
